# Skin-Inspired Tactile Sensor on Cellulose Fiber Substrates with Interfacial Microstructure for Health Monitoring and Guitar Posture Feedback

**DOI:** 10.3390/bios13020174

**Published:** 2023-01-22

**Authors:** Rajat Subhra Karmakar, Chia-Pei Chu, Chia-Lin Li, Chun-Hway Hsueh, Ying-Chih Liao, Yen-Wen Lu

**Affiliations:** 1 Department of Biomechatronics Engineering, National Taiwan University, 10617 Taipei, Taiwan; 2 Department of Chemical Engineering, National Taiwan University, 10617 Taipei, Taiwan; 3 Department of Materials Science and Engineering, National Taiwan University, 10617 Taipei, Taiwan

**Keywords:** skin-inspired, tactile sensors, screen printing, electrical contact resistance, vital sign monitoring, remote learning

## Abstract

Skin-inspired flexible tactile sensors, with interfacial microstructure, are developed on cellulose fiber substrates for subtle pressure applications. Our device is made of two cellulose fiber substrates with conductive microscale structures, which emulate the randomly distributed spinosum in between the dermis and epidermis layers of the human skin. The microstructures not only permit a higher stress concentration at the tips but also generate electrical contact points and change contact resistance between the top and bottom substrates when the pressure is applied. Meanwhile, cellulose fibers possessing viscoelastic and biocompatible properties are utilized as substrates to mimic the dermis and epidermis layers of the skin. The electrical contact resistances (ECR) are then measured to quantify the tactile information. The microstructures and the substrate properties are studied to enhance the sensors’ sensitivity. A very high sensitivity (14.4 kPa^−1^) and fast recovery time (approx. 2.5 ms) are achieved in the subtle pressure range (approx. 0–0.05 kPa). The device can detect subtle pressures from the human body due to breathing patterns and voice activity showing its potential for healthcare. Further, the guitar strumming and chord progression of the players with different skill levels are assessed to monitor the muscle strain during guitar playing, showing its potential for posture feedback in playing guitar or another musical instrument.

## 1. Introduction

In recent years, the scientific community has witnessed a rapid evolution of flexible electronic devices; among them, tactile sensors are gradually becoming the most important component for applications in healthcare, education and environmental sectors [1,2,3,4,5]. Especially in the healthcare sector, flexible tactile sensors have made great advancements in the continuous monitoring of vital physiological parameters (i.e., respiratory rate, heart rate, etc.) by enabling the integration of wearable electronics with the human body [5,6,7,8,9]. In education, tactile sensors have just begun to be used to help students track their learning progress or monitor their postures when playing instruments [10]. Many applications in these sectors require the sensors to have high sensitivity to differentiate tiny movements of the muscles in the subtle pressure region (approx. 0–0.5 kPa), or to respond to complicated dynamic stimuli, mostly alternating and cyclic loads [11,12,13].

The technological inspirations for the highly-sensitive tactile sensor design often resort to Mother Nature, since she can provide scientific solutions. The skin, as the largest organ of the human body, is capable of magnificent sensory functions because of its special structures, including epidermal–dermal hill-shaped structures, various mechanoreceptors and afferent nerves [14,15,16]. All these microstructures and receptors enable human skin to simultaneously perceive and differentiate between multiple tactile stimuli. These special attributes of human skin have inspired the scientific community to develop wearable tactile sensors that employ the microscale structure of the dermis or epidermis layer [6,9,17,18,19,20,21,22,23]. They mostly employ complicated microstructures and interface layers. Therefore, it is highly necessary to implement the skin-inspired structure by using a simple and industry-compatible process that results in a high sensitivity suitable for subtle pressure detection.

When it comes to tactile sensors, there are several types present in the market, which are categorized as resistive [12], piezoelectric [24], capacitive [25] and optical [26], based on the sensing mechanisms. Due to low cost and simple fabrication, the resistive tactile sensor is one of the most common and widely applicable techniques [27,28,29]. Their key mechanism evolves around either (1) the resistance change caused by the change of sensor geometry [29,30,31] or (2) the electrical contact resistance (ECR) change between two conductive layers under the applied forces [31,32,33,34,35,36,37]. The latter has proven to be highly effective and to have a good dynamic response, a high sensitivity and a tunable working range [31]. ECR is represented by the resistance between contact surfaces; the variation of ECR occurs when the contact conditions and areas change after the application of external pressures.

Inspired by the skin perception mechanism, this work proposes a highly sensitive ECR-based tactile sensor for subtle pressure detection by implementing the microstructures of the dermis layer with a novel sensing mechanism. The microstructures, screen printed with conductive inks, emulate the randomly distributed spinosum in between the dermis and epidermis layers of human skin. They not only concentrate higher stress at the tips, but also generate electrical conduct points and change electrical contact resistance (ECR) between the top and bottom substrates when the pressure is applied. Meanwhile, cellulose fibers, thanks to their unique viscoelastic and biocompatible properties, are employed as substrates and to mimic the dermis and epidermis layers of the skin. The electrical contact resistances (ECR) are then measured to quantify the tactile information. Further, a systematic investigation of the impact of both sensing microstructures and sensor substrates is needed. The influence of the cellulose fiber substrates over the tactile sensing performance in their elasticity, compressibility and porosity are studied and optimized. Our sensors can achieve high sensitivity in the subtle pressure region (e.g., approx. 0–0.5 kPa), and can be applicable in wearable applications, including vital sign detection and posture recognition.

## 2. Materials and Methods

### 2.1. Contact Resistance at Interfaces

The ECR-based tactile sensor and its mechanism have been previously reported by our group [35,36,37]. In short, electrical contact resistance (ECR) is a microscopic phenomenon that is represented by the resistance between two conductive surfaces. The contact resistance can be reduced by the generation of electrical contact points at the interface between two conductive surfaces when an external pressure or force is applied. Thus, the tactile force or pressure can be measured by monitoring the contact resistance variations.

The relationship between ECR and surface contact area can be calculated by using fractal geometry through structure function, which can be defined as a statistical ensemble physical representation of the mean square of the difference in height expected over any spatial distance [35,38,39,40]. Hence, based on the references, the relationship between the contact resistance (R) and external Pressure (P) can be represented by the following equation [35,38,39]:

(1) R=AaΓG(D−1)LDλ(DE(2−D)P)D2

where A_a_ is the identifiable contact area of the interface, Γ is a constant related to the actual contact area of conductive surfaces, G is a scaling constant that can be denoted as the non-dimensional roughness parameter, D is the self-similar fractal dimension and λ is the effective electrical conductivity of the contact surface, whereas P is the external pressure and E is the elasticity.

After considering the material properties of bulk modulus and compressibility, the equation (1) can be rewritten as [35,38,39]:

(2) R=AaΓG(D−1)LDλ(D(2−D)3ΔV/V0(1–2ν))D2

where compressibility of the material is represented by the relative volume change (∆V/V_0_) to pressure P and ν is the Poisson ratio. Equations (1) and (2) describe the contact resistance at an interface as a function of the surface topography and mechanical properties of the substrates (e.g., elasticity, compressibility, etc.).

### 2.2. Device Fabrication and Assembly

**Figure 1:**
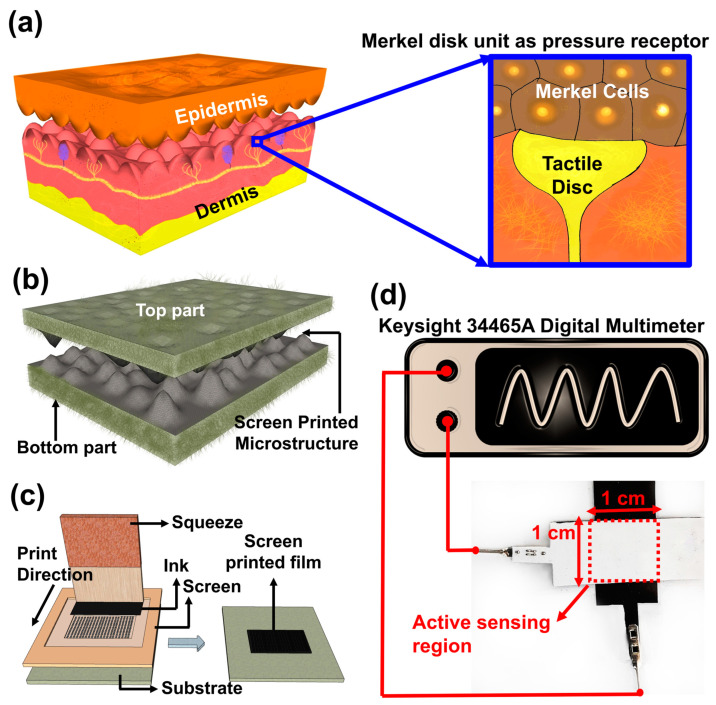
(**a**) Illustration of the biological microscale structures of the human epidermis; the underlying spinosum of the epidermis layer is a crucial element for high sensitivity. To respond to tactile stimulation, there are primary pressure receptors, called Merkel disc, at the tip around the microstructures (**b**) our tactile sensor had a top and a bottom substrate; they were assembled face-to-face, with screen-printed microstructure in between at the interface (**c**) schematic diagram of screen printing method (**d**) optical image of the fabricated tactile sensor along with the schematic diagram of the measurement process. The fabricated sensor had an active sensing area of 1 × 1 cm^2^, where the pressure was applied.

As the largest sensory organ in the human body, the skin perceives and distinguishes external stimuli (e.g., pressure, touch, bending, stretching and textures). For its tactile information perception, the skin mainly relies on the mechanoreceptors around the microstructures in between the epidermis and dermis layers, as shown in Figure 1a [41,42,43]. The outermost layer of the epidermis, with a high elastic modulus, provides the skin with toughness, whereas the dermis layer, with a low elastic modulus collagen, has sensory receptors [14,44,45]. In between the epidermis and dermis layers, lies the densely distributed microscale structures called stratum spinosum, which can produce a high and local stress concentration at the microstructure tips near receptors [21,46,47]. In particular, to respond to and transmit the sensation of light touch and low-frequency vibration, there is a group of tactile mechanoreceptors, primarily Merkel disc, at the tip around the microstructures. Inspired by this tactile perception mechanism, the microstructures are adapted to our sensors.

Figure 1b depicts our concept to have this skin-inspired tactile sensor, which consists of two substrates and was assembled face-to-face, with the microstructures in between. Cellulose fiber substrates, which function similarly to the epidermis and dermis layers of the skins, with different textures and mechanical properties, were used and denoted by S1 to S4, including a standard photocopy paper (S1), cover paper (S2), laboratory tissue paper (S3) and paper towel (S4). A relatively rigid polyethylene terephthalate (PET) substrate with a smooth surface was used as the control sample (CS), to which the cellulose fiber samples were compared.

Two different types of carbon-based conductive inks, termed Ink1 and Ink2, were used as the sensing material. Ink1 was a graphene-based water-insoluble conductive ink (Graphene Ink, Euflex Corporation Ltd., New Taipei City, TW) whereas Ink2 was a carbon-black and graphite-based water-soluble conductive ink (Electric Paint, Bare Conductive, London, UK). Both inks were employed over the substrates by using the screen printing method, as shown in Figure 1c. Once the inks were printed and cured, the substrates were cut and combined face-to-face, followed by a careful encapsulation with a commercially available adhesive PET. The encapsulation was done in such a way that the surfaces of the screen-printed substrates touched each other without exerting any extra force. The formed tactile sensing device had an effective sensing area with a dimension of 1 cm × 1 cm, where the pressure was applied. The photograph of the assembled tactile sensor with measurement setup was shown in Figure 1d.

### 2.3. Characterization Methodologies

The microstructures in the sensors (e.g., the screen-printed films with Ink1 or Ink2) were characterized by their properties. Their mechanical properties in elastic modulus and hardness were investigated by using the nanoindenter (TI 950 TriboIndenter, Hysitron, Minneapolis, MN, US) using a Berkovich 142.3° diamond probe at a constant indentation depth of 150 nm. The average surface roughness values were investigated by using a DektakXT-M stylus profilometer (Bruker, MA, USA). The chemical properties were characterized by using UniDRON microscopic Raman/PL spectroscopy (CL Technology Co., Ltd., New Taipei City, Taiwan).

The cellulose fiber substrates were also characterized. Their mechanical properties in elastic modulus and compressibility were tested by using an MTS 42.503 Static Tensile Testing Machine (MTS Criterion 42.503 Test System, Eden Prairie, MN, USA). Their deformation under subtle pressure was investigated by analyzing the indentation depths under the exerted load by the same nanoindenter system. The morphological analyses were executed by using a field emission scanning electron microscope (FESEM) (Hitachi S-4800, Hitachi HighTechnologies Corp., Tokyo, Japan). Their porosity was determined by Accupyc II 1340 Pycnometer (Micromeritics, Norcross, GA, USA).

The electrical characterization of all fabricated tactile sensors was carried out at room temperature to investigate the ECR variation with applied pressure. The schematic diagram of the measurement setup was presented in Figure 1d. Tactile sensors were carefully attached to a custom-made platform and adhesive tape was used for the attachment to eliminate any multidirectional shear force. To apply the pressure, precision balance weights were used from a vertical direction and the pressure range was limited to 1 kPa. A Keysight 34465A digital multimeter (Keysight, Santa Rosa, CA, USA) was used to monitor the change in electrical contact resistance. The repeatability and endurance tests were carried out by subjecting the tactile sensors to a continuous 2000 cycles of 1 kPa pressure. For this purpose, a JSV-H1000 vertical stand equipped with an ALGOL force gauge (ALGOL Instrument Co., Ltd., Taoyuan, Taiwan) was used.

Furthermore, the tactile sensors were tested for two wearable applications: (i) to monitor vital signs and (ii) to distinguish the strumming patterns and chord progression of a musical instrument (guitar). In the first application, the sensors were packaged and attached to a cotton wrap and worn around the throat to detect the vibrations during speaking. The sensors were further attached to the chest to detect breathing patterns such as eupnea and tachypnea. In the second application, the sensors were attached to an arm wrap and worn on the forearm and elbow to detect the strumming patterns of guitar playing. The sensors were worn on the left hand to detect the chords that candidates played during testing. All these wearable tests were performed with the consent of the candidates and the experiment was approved by the Research Ethics Committee of National Taiwan University.

## 3. Results

### 3.1. Properties of Carbon Inks

**Figure 2:**
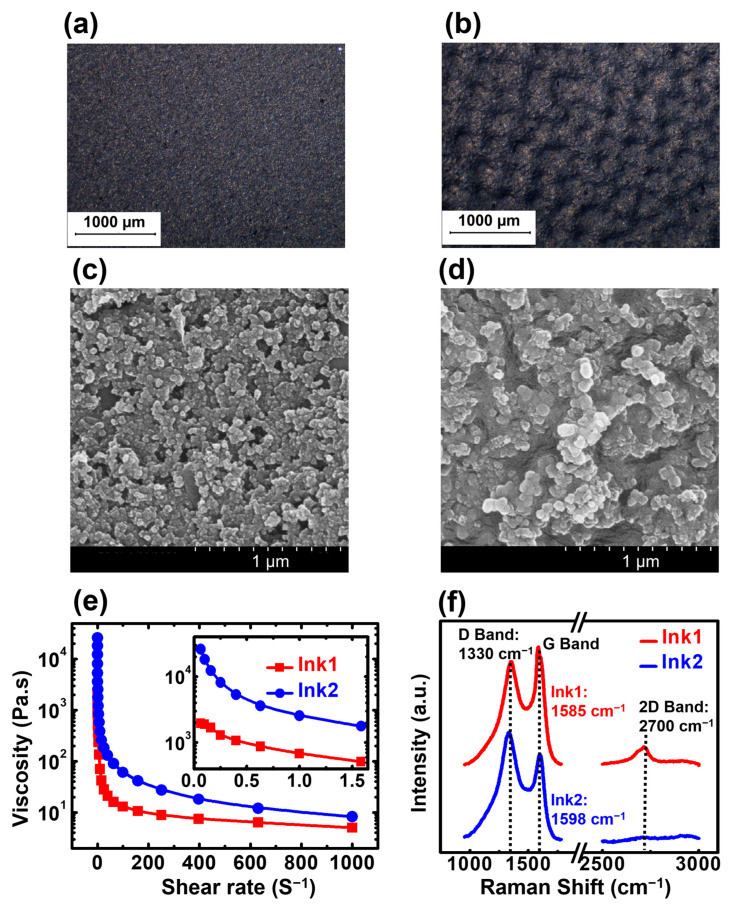
Optical microscope image of (**a**) Ink1 and (**b**) Ink2 over a glass substrate. SEM images of (**c**) Ink1 and (**d**) Ink2 over a glass substrate (**e**) viscosity data of Ink1 and Ink2 for the shear rate range of approx. 0–1000 S^−1^. The inset figure shows the viscosity data for the shear rate range of approx. 0–1.5 S^−1^ (**f**) Raman spectra of Ink1 and Ink2 showing the material composition of both inks. The D, G and 2D bands were explored to understand the presence of graphene layers.

Optical microscope images with the 1000 µm scale bar of screen-printed inks over glass substrate are presented in Figure 2a,b, which give a primary understanding of film morphology. Figure 2a shows the morphology of the film formed by using Ink1, which appears to be smoother than the Ink2 film as shown in Figure 2b. Compared to Ink1 film, the Ink2 film possessed multiple hill-shaped microstructures, which led to a higher surface roughness. This statement was further confirmed by measuring the rms surface roughness of Ink1 and Ink2 films by using the surface profiler, which was recorded as 0.634 µm and 0.999 µm. The average thicknesses of both films were further reported as 12 µm and 33.2 µm, respectively, by the surface profiler also. Figure 2c,d show the magnified SEM image of both films with the scale bar of 1 µm, where the clusters formed by the carbon particles mixed with the binders can be observed. The smallest clusters have an average size of 50 nm and 96 nm for Ink1 and Ink2, respectively. It can also be observed in Figure 2d that the clusters were arranged in a hill-valley-shaped microstructure formation for Ink2 films, whereas for Ink1 films they formed a relatively flat plateau, as shown in Figure 2c.

The rheological characteristics of carbon inks are presented in Figure 2e. The viscosity of both Ink1 and Ink2 were investigated for the shear rate of 0 to 1000 S^−1^. Ink2 had a higher viscosity than Ink1 for the entire shear rate range. At the shear rate of 1 S^−1^, the viscosity of Ink1 was reported as 682.888 Pa.S, whereas for Ink2 this value was 2549.14 Pa.S. The high viscosity of Ink1 and Ink2 was predominantly the main reason behind the difference between the film thickness and surface properties of Ink1 and Ink2 films, which played a key role in device performance.

The chemical properties of both carbon inks were investigated by using Raman spectroscopy and the data is presented in Figure 2f. There were two primary peaks that were observed for Ink1 and Ink2. The D bands with high intensity were observed at 1330 cm^−1^, which is identical for both Ink1 and Ink2. This band at a lower wavelength was usually ascribed to sp3-hybridized carbon atoms that remain in a disordered state [48]. However, the G band of Ink1 was observed at 1585 cm^−1^, whereas for Ink2 the location of the G band was found at the higher wavelength of 1598 cm^−1^. In general, the G band was ascribed to the sp2-hybridized carbon atoms and a shift at a higher wavenumber was attributed to the shorter bond length [48,49]. For Ink1, the location of the G band was not only located at the higher wavenumber, but also possessed a much sharper feature that was attributed to sp2 phonon vibrations, confirming the presence of graphene in the polyester binder [50,51,52]. Moreover, at a 2700 cm^−1^ wavelength, an additional 2D band was observed for Ink1 film, which is an indicator of the number of graphene layers [52]. Here, the band appeared to be broadened, attributed to the fact that the graphene ink film contains a few layers with some defects.

The ratio of ID (intensity of D band) and IG (intensity of G band) were further calculated for both Ink1 and Ink2 to understand the degree of graphitization [52,53,54,55]. The ID/IG values for Ink1 and Ink2 were reported as 0.83 and 1.25. Ink2 had a higher value of ID/IG ratio, which showed the presence of turbostratic carbon and disordered surface structures [54]. However, Ink1 had a comparatively lower value of ID/IG, implying a higher degree of graphitization, which also led to a relatively higher electrical conductivity [55]. This statement was further confirmed by sheet resistance data obtained by a four-point probe measurement. The sheet resistance of Ink1 film was reported as 90939.5 Ω/sq, which is lower than the sheet resistance value of Ink2, i.e., 92163.9 Ω/sq.

Moreover, the elastic modulus and hardness of Ink1 and Ink2 films were also investigated by using the nanoindenter. At least five samples for both inks (Ink1 and Ink2) were tested under the constant indentation depth of 150 nm and the elastic modulus and hardness values were directly recorded. For Ink1, the reported elastic modulus value was 4.417 GPa along with a 0.094 GPa of hardness. Meanwhile, the elastic modulus and hardness values for Ink2 were 5.324 GPa and 0.151 GPa. Hence, Ink2 was not only more viscous than Ink1, but the film formed by it possessed higher hardness values than Ink1 films. This also led to relatively a higher rigidity for Ink2 films. All these parameters are recorded in Table 1.

### 3.2. Morphological Analysis of Screen-Printed Substrates

**Figure 3:**
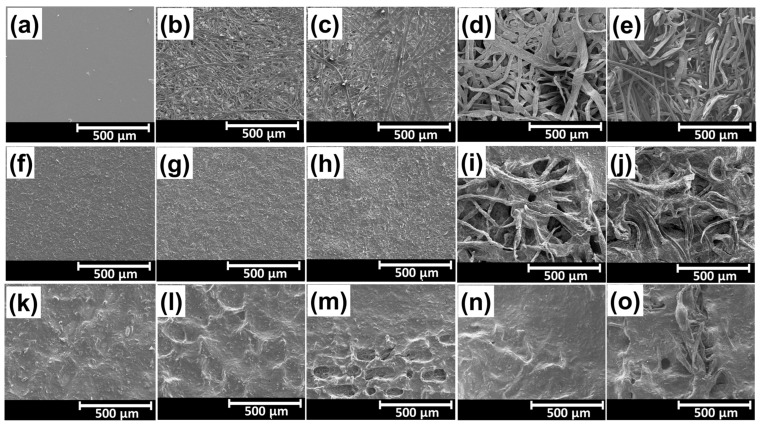
SEM images of all five substrates (e.g., CS, S1, S2, S3 and S4) with different surface conditions. (**a**), (**b**), (**c**), (**d**), (**e**) the substrates without ink coating (**f**), (**g**), (**h**), (**i**), (**j**) the substrates with Ink1 coating (**k**), (**l**), (**m**), (**n**), (**o**) the substrates with Ink2 coating.

Figure 3 shows the scanning electron microscopy (SEM) images of the pristine and coated substrate samples for the sensors. The CS substrate had a smooth surface, whereas others (e.g., S1–S4) had microscale textures with fibrous structures, as presented in Figure 3a–e. S1 and S2 substrates, which consisted of a compact network of cellulose fibers, showed relatively rough and porous surfaces. S3 and S4 substrates had loosely distributed larger fibers, where S4 had a more loosely distributed cellulose fiber network, leading to higher porosity and surface roughness, compared to the S3 substrate., etc. Figure 3f–j demonstrates the SEM images of the substrates coated with Ink1. Figure 3f,g show the Ink1-coated surface of the CS and S1 substrates. Owing to the smooth nature of the PET substrate, the screen-printed film’s surface turned out to be similar. Whereas for S1, the screen-printed film became rougher than the film formed over CS, owing to the fibrous structure at the surface. A wavier and rougher surface was obtained for the film formed over the wavy surface of S2, as shown in Figure 3h. Meanwhile, Figure 3i,j show the highly fibrous and porous structure of S3 and S4 substrates that were minutely covered with Ink1. Owing to the lower viscosity, Ink1 could not only cover every fiber of the paper substrate, but also mimic the substrate’s original features and texture.

**Figure 4:**
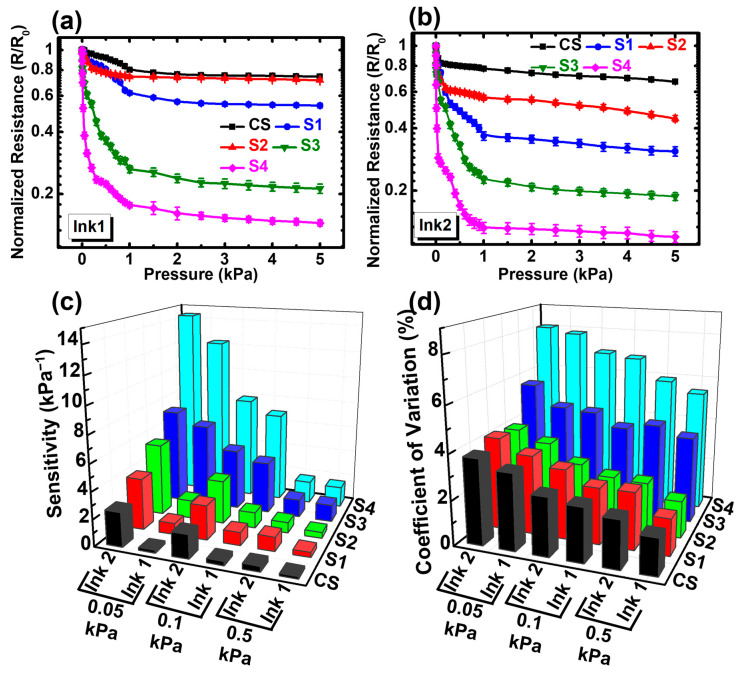
Electrical characterization data of paper-based tactile sensors. Normalized resistance (R/R_0_) changes with applied pressure for sensors with (**a**) Ink1 and (**b**) Ink2. The sensitivity and coefficient of variation data are also presented in (**c**) and (**d**), respectively.

The SEM images of Ink2 coated substrates are presented in Figure 3k–o. The hill-valley-shaped rough surface was obtained for CS and S1, as shown in Figure 4k,l, owing to the higher intrinsic roughness value of Ink2. The roughness of Ink2 film over S1 was higher compared to CS, owing to the fibrous structure of S2. Figure 3m shows that the film formed over the wavier part of the S2 had a much rougher surface texture compared to the film formed over the flatter parts. Meanwhile, Figure 3n,o demonstrate that Ink2 completely covered the highly fibrous and porous structure of the lab tissue paper (S3) and paper towel (S4). Ink2 barely mimicked the surface characteristics of substrates, owing to the higher viscosity. However, the texture of substrates still played an important role, i.e., the Ink2 film became rougher for uneven surfaces. Meanwhile, it is evident that the fibrous structure of S3 and S4 substrates coated with Ink1 and Ink2 resembled the randomly distributed spinosum layer between the dermis and epidermis layer, which could lead to a better sensing characteristic. The intrinsic surface properties of substrates will have an impact on the device characteristics since the substrates’ morphology directly impacts the sensing layer’s properties.

### 3.3. Electrical Characterization

The sensing characteristics of all skin-inspired tactile sensors with Ink1 and Ink2 are presented in Figure 4. The characterization was done at room temperature and at least six (6) samples were measured to investigate the changes in normalized electrical contact resistance (ECR) with applied pressure. The normalized ECR was defined as the ratio between the ECR at the specific pressure to the ECR at zero pressure, i.e., R/R_0_. The results of the sensors with Ink1 and Ink2 are graphically presented in Figure 4a,b. The applied pressure range for this experiment was extended to 5 kPa. All the fabricated sensors exhibited distinct characteristics at lower (0–1 kPa) and higher (1–5 kPa) applied pressure. The response at lower pressure (0–1 kPa) is separately presented in Supplementary Figure S1. The normalized ECR values of the samples fabricated with Ink2 were much lower than Ink1 at both higher and lower pressure, which can be attributed to the rougher surface of Ink2 films. The control sample (CS) with Ink1 showed almost a flattened line at the pressure range approx. 0–0.5 kPa compared to CS with Ink2, since the rougher surface resulted in a curved line in the graph, as shown in Supplementary Figure S1. A relatively larger change in normalized resistance was observed for S1 and S2 samples with both Ink1 and Ink2 because of the rougher surface texture of the sensing layer formed over paper substrates. It was observed that the normalized ECR of S1 was higher at lower pressure compared to S2 for both inks. Meanwhile, the ECR values of S1 became lower than S2 at a pressure higher than 0.5 kPa and 0.2 kPa for Ink1 and Ink2, respectively, due to better compressibility.

However, the largest change in normalized ECR was observed for the sensors with S3 and S4 for both inks. Both samples showed rapid changes at approx. 0–0.1 kPa and the normalized resistance value was much smaller than for CS, S1 and S2. At 0.1 kPa, a 40% change in normalized ECR was observed for the sensor with S3, whereas an almost 70% change was observed for the sensor with S4 due to the microstructures that mimic the randomly distributed spinosum layer in the dermis. Although the fibrous surfaces of S3 and S4 were totally covered with Ink2, they could still replicate a similar microstructure, owing to the intrinsic higher roughness of Ink2. Moreover, the further change in normalized resistance at higher pressure (1–5 kPa) was caused by fibrous and porous structures of the substrate surfaces combined with the higher compressibility.

The sensitivity of tactile sensors was calculated from the resistance-pressure data with the following equation:

S = (ΔR/R_0_)/∆P (3)

where S is the sensitivity, ΔR is the ECR difference, R_0_ is the initial resistance at zero pressure and ΔP is the pressure difference. The sensitivity of the sensors with Ink1 and Ink2 is demonstrated in Figure 4c. All the sensors showed a higher sensitivity at 0.05 kPa pressure, proving that the fabricated sensors are suitable for subtle pressure applications. The sensor with Ink2 showed a higher sensitivity than the one with Ink1, owing to the larger change in normalized ECR. The surface roughness of Ink2 was higher than Ink1, which led to a higher original ECR value. Figure 4c also shows that the sensitivity increased for the substrates with higher compressibility and surface roughness. Both S3 and S4 tactile sensors exhibited the largest sensitivity compared to all, since both of these substrates had high compressibility, porosity and microscale fibrous structures. The porous substrates coated with conductive inks perfectly generated the randomly distributed microstructure at the interface, which truly mimicked the dermis and epidermis layer resulting in high sensitivity caused by ECR variation as shown in Figure 4c. Hence, the highest reported sensitivity of this work is 14.4 kPa^−1^ and 12.3 kPa^−1^ for the S4 tactile sensor with Ink2 and Ink1, respectively, at 0.05 kPa, proving that these sensors can be an ideal candidate for applications in subtle pressure regions.

In addition, the coefficient of variation (CV) of fabricated sensors was calculated and presented in Figure 4d. The coefficient of variation can be defined as the ratio of the standard deviation and the mean value of normalized ECR. This is a standardized indication that shows the extent of stability and repeatability of skin-inspired tactile sensors. To calculate the CV, more than 20 samples of each tactile sensor were measured. The CV was low for the samples with rigid substrates (e.g., CS, S1, and S2), but higher for the samples with porous and compressible substrates (e.g., S3 and S4). This shows a clear correlation between the device’s stability and mechanical properties. The increment in CV values for S3 and S4 means that the sensors with compressible substrates could suffer from data instability. This trend was similar for all pressure ranges. However, the CV was significantly high at 0.05 kPa applied pressure, which shows that, at lower pressure, data variation is higher than the lower pressure. This trend indicates that the electrical current conduction at the interface was more stable with higher pressure, since the conduction point generation with applied pressure will be more compared to lower pressure.

**Figure 5:**
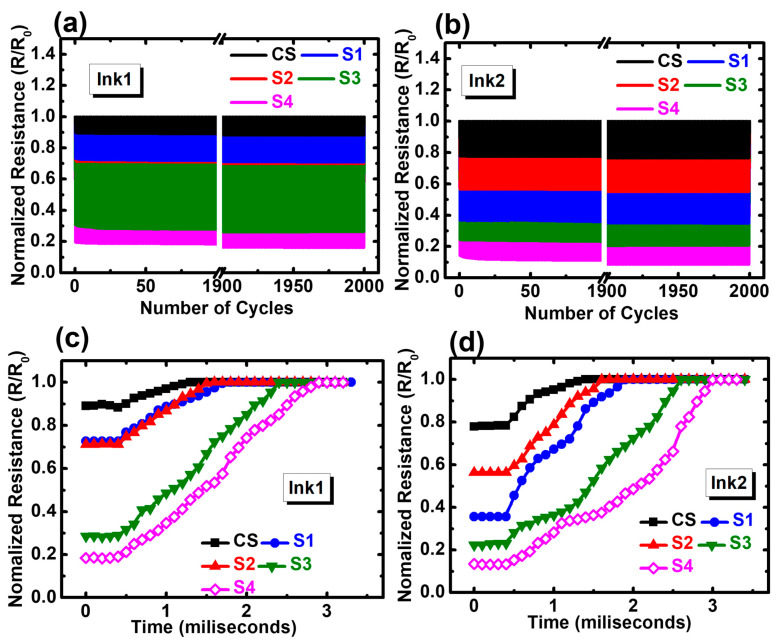
Reversible testing for 2000 cycles of repeated loading and unloading of 1 kPa applied pressure for sensors with (**a**) Ink1 and (**b**) Ink2. The time-dependent resistance characteristics for tactile sensors with (**d**) Ink1 and (**e**) Ink2.

The dynamic reversible testing of tactile sensors for 2000 loading/unloading cycles with 1 kPa applied pressure was performed to confirm the repeatability and durability of fabricated tactile sensors. The cycling characteristics are shown in Figure 5a,b. The first and last 100 cycles of the entire 2000 cycles were plotted and a steady repeatable response from all the fabricated sensors was observed. However, a slight deviation from the initial normalized ECR was observed in the last 100 cycles. The deviation was higher for the tactile sensors with Ink2, which implies the rigid Ink2 film suffers from micro-cracks after 2000 cycles of loading and unloading of pressure. However, the substrates with a higher elastic modulus seemed more stable, compared to the substrates with higher compressibility, i.e., the normalized ECR deviation (ΔECR) was higher for the tactile sensors with S3 and S4 compared to others. The ΔECR can be expressed by the difference between the mean ECR value at the first 100 cycles and the last 100 cycles. This deviation in normalized ECR data from Supplementary Figure S2 clearly shows that the tactile sensors with softer substrates with high porosity (S3 and S4) with Ink2 had larger ECR deviation, which means that, to obtain a stable response over a longer period of time, a substrate with a higher elastic modulus can be a better choice.

The recovery time of every tactile sensor for a single cycle was also investigated and plotted in Figure 5c,d. The time required for each tactile sensor to reach the high resistive state after releasing the pressure was termed as the recovery time, and it can be understood that the tactile sensors with higher compressibility and porosity (S3 and S4) required a longer recovery time when pressure was released, since softer substrates can be deformed easily and require a longer time to come back to the original state compared to the substrates with higher elastic modulus. The longest recovery times reported were 2.4 and 2.5 ms, and they were recorded for the sample with S4 substrate coated with Ink1 and Ink2, respectively. The recovery time for all the samples was presented in Supplementary Figure S2. This data proves that the tactile sensors with higher compressibility may suffer from data deviation and a longer recovery time, despite showing ultrahigh sensitivity at subtle pressure regions. Thus, careful optimization is highly recommended to select the right substrate material with high sensitivity and relatively lower recovery time for future wearable applications.

### 3.4. Theoretical Model and Physical Analysis

**Figure 6:**
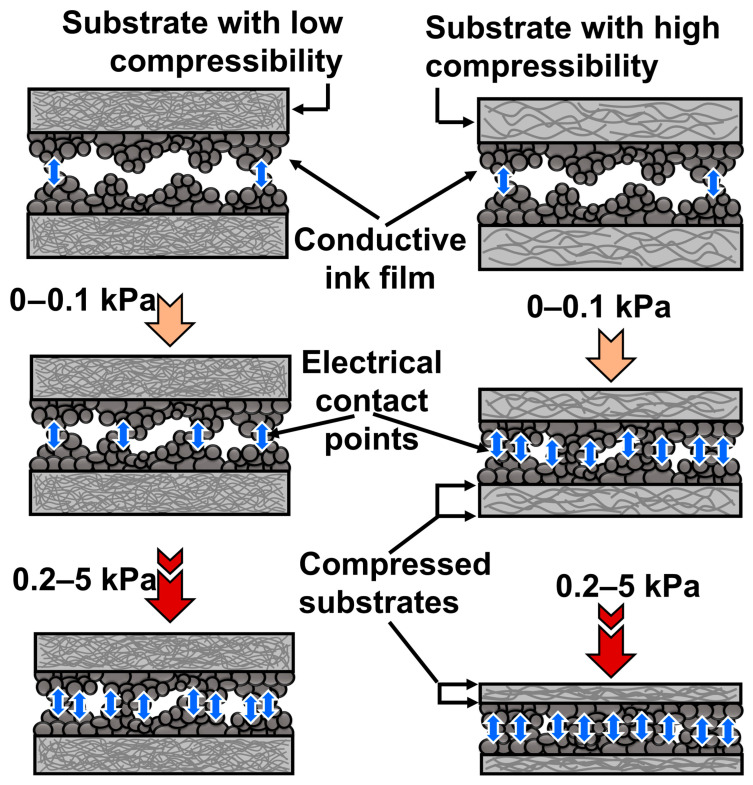
Schematic diagram to show the ECR variation mechanism for our tactile sensors with the substrates in different porosity/compressibility. The arrow symbolizes the electrical contact points through which electrical conduction occurs. Compression of cellulose fiber substrates under applied pressure also can be observed in this figure.

The key mechanism for our tactile sensors is the electrical contact resistance (ECR) variation represented in Figure 6. The porosity-induced compressibility and its impact on ECR variation were demonstrated. Similarly to the randomly distributed spinosum layer between the dermis and epidermis layer, the microstructure at the interface of the presented skin-inspired tactile sensor is responsible for tactile sensing application. By performing the face-to-face assembly of screen-printed substrates, the tactile sensor was formed, which mimics the epidermis and dermis layer of human skin. The electrical current can pass through the contact points generated at the interface with the microstructure. The conduction points are relatively less for rougher surfaces, resulting in higher ECR values. The ECR value gradually drops with increasing pressure, owing to the generation of conduction points at the interface. This means that the micro-scaled conductive ink cluster can act like the pressure receptive unit, which is similar to the Merkel cells present in human skin.

**Figure 7:**
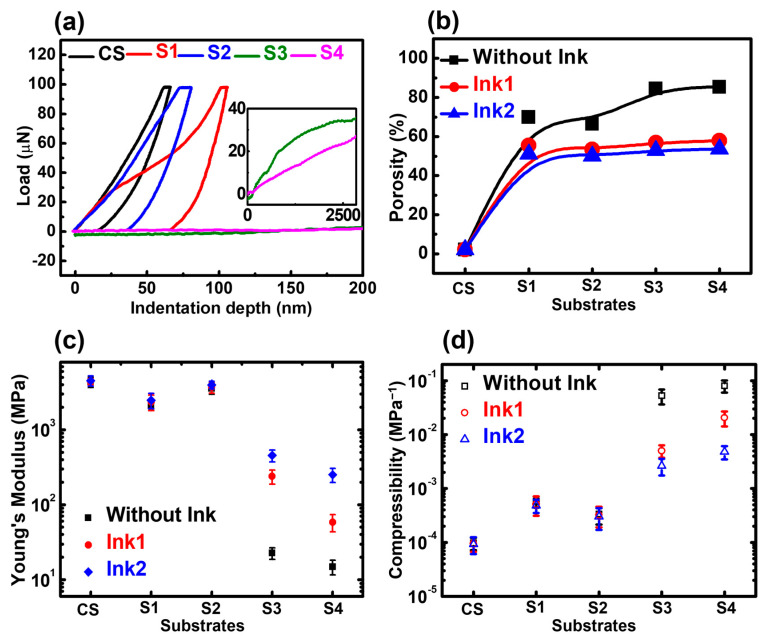
(**a**) Nanoindentation data of our cellulose fiber substrates (**b**) porosity values of cellulose fiber substrates determined by helium pycnometry (**c**) elastic modulus and (**d**) compressibility data of four cellulose fiber substrates and PET control sample (CS) with and without coating of Ink1 and Ink2.

Two different types of cellulose fiber substrates, which have low and high porosity/compressibility, are shown in Figure 6. The substrates with low porosity and compressibility (CS, S1 and S2) generate relatively fewer electrical contact points at the interfaces compared to the substrates with high porosity and compressibility (S3 and S4). The physical properties of the substrates were confirmed by nanoindentation, pycnometry and a tensile stress-strain experiment. Figure 7a shows the nanoindentation data of our cellulose fiber substrates (S1–S4), along with the PET substrate as the control sample (CS). The load by the Berkovich 142.3° diamond probe over the substrate material was plotted as a function of the indentation depth. For CS, the exerted load reached the maximum value of 98 µN at the indentation depth of 63.4 nm. Meanwhile, the recorded indentation depth of S1 and S2 were 103.2 nm and 75.1 nm, respectively, for 98 µN applied load. However, for lab tissue paper substrate (S3) and paper towel (S4), the indentation depth can surpass 2500 nm and the recorded loads were 35.7 µN and 27.1 µN at 2850 nm indentation depth. There was no typical unloading curve for S3 and S4, showing both to have higher displacement under low pressure, which could be related to the higher porosity of the substrates.

To obtain the porosity data, the cellulose fiber substrates were sealed inside a pycnometer and the helium gas was used for measuring the fiber volume. The porosity of the cellulose fiber substrates was calculated by this equation [56]:

(4) $$\mathrm{Porosity}=1-\frac{V_{f}}{V_{T}}$$

where V_f_ is the volume of cellulose fibers measured by pycnometer and V_T_ can be denoted as the total volume of the cellulose fiber sample used for pycnometry, which can simply be calculated by multiplying the length, width and thickness. The calculated porosity values of cellulose fiber substrates are presented in Figure 7b. The PET control sample had the lowest amount of porosity, i.e., 2.15%. However, photocopy paper (S1) had 70% porosity, which is higher than the reported porosity of cover paper (S2), i.e., 66%. Meanwhile, both lab tissue paper (S3) and paper towel (S4) exhibited very high porosity, i.e., 84% and 85%, respectively. From Figure 6b, it also can be understood that the porosity of cellulose fiber substrates reduced after screen-printing, which proves the absorbance of inks at the porous surface of cellulose fiber substrates.

Once the porous nature of cellulose fiber substrates was confirmed, the mechanical properties (elastic modulus and compressibility) were investigated by performing the tensile stress-strain test. All the samples were cut into 1 cm wide and 5 cm long pieces. They were clamped at both sides in an MTS 42.503 static tensile testing machine and the tensile force was applied until the samples tore at the middle section. The elasticity of substrates with and without inks is presented in Figure 7c. The CS possesses the elasticity of 4332 MPa in a pristine situation, while it has 4409 MPa and 4524 MPa, respectively, with Ink1 and Ink2 coating—implying no major influence over CS’s elasticity. A similar trend was observed for substrate S1 (photocopy paper) and S2 (cover paper), as the value of E was close for the pristine state and coated state. However, a more drastic change in E was observed for S3 (lab tissue paper) and S4 (paper towel). At pristine state, the reported values of E for S3 and S4 were 22 MPa and 14 Mpa, which belong to the same range of the elastic modulus of skin (4.6 MPa–22 MPa) [57]. For S3, the value of E increased to 238 MPa after applying Ink1, whereas, for Ink2, the elastic modulus value was reported as 454 MPa. Meanwhile, for S4, the increased elastic modulus values were 58 MPa (Ink1) and 250 MPa (Ink2). Since S3 and S4 had better absorbability, which is contributed to by their highly porous nature as confirmed before, their properties also changed drastically after coating with Ink1 and Ink2.

Figure 7d shows the compressibility of the substrates with and without the coating of Ink1 and Ink2. CS, S1 and S2 substrates possessed similar compressibility in both pristine and coated states. S3 and S4 had higher compressibility compared to others, which can be attributed to the higher porosity percentage of both substrates, implying both substrates have higher deformation after applying the pressure, which directly impacts the tactile sensing characteristics. In addition, S3 and S4 had smaller compressibility values after coating with Ink1 and Ink2. Particularly, Ink2-coated substrates had lower compressibility, since Ink2 inherently possesses a higher elasticity compared to Ink1, as confirmed by the nanoindentation experiment.

Hence, based on the physical analysis data, it can be understood from Figure 6 that the deformation was negligible for CS, S1 and S2 substrates under lower pressure, i.e., 0–0.1 kPa, which means the surface roughness plays the key role in device performance. This results in a lower number of electrical contact points at the interface between two printed substrates. However, for highly compressible substrates (S3 and S4), the deformation was more significant at the lower pressure range, and, by coupling with surface roughness phenomena, the electrical contact point generation at the interface was escalated. Hence, the tactile sensors formed over a porous and highly compressible substrate (S3 and S4) showed a steep response curve before 0.1 kPa, as the subtle pressure was able to compress the porous substrate, leading to a much higher sensitivity reported in Figure 4c in the subtle pressure region (14.4 kPa^−1^ at 0.05 kPa) compared to the sensors with CS, S1 and S2.

At higher pressure (approx. 0.2–5 kPa), the electrical contact point generation became more significant compared to lower pressure for all substrates, as shown in Figure 6. However, the deformation was more severe for S3 and S4 substrates compared to CS, S1 and S2, which led to even more electrical contact point generation at the interface. It can be understood that the substrates with dense cellulose fiber possess lower porosity. The deformation of pores was less under applied pressure, which resulted in lower compressibility and fewer electrical contact points at the interface, whereas for porous substrates, the cellulose fibers were loosely distributed and the interconnected pores could be easily deformed under applied pressure. Hence, the porous substrate exhibited higher compressibility and deformability, which resulted in the generation of more electrical contact points at the interface at higher pressure.

In addition, the substrates with higher compressibility showed slower recovery time compared to the substrates with lower compressibility, owing to the time required to return to the original decompressed state. For less compressible substrates (CS, S1, and S2), only interface layers were involved for ECR variation; a rapid recovery time could be obtained for harder substrates, since the interfacial gap increased quickly after releasing the pressure. Hence, it can be understood that, with the right optimization of substrate material, both high sensitivity and faster response can be achieved for tactile sensors.

### 3.5. Demonstration of Wearable Applications

#### 3.5.1. Vital Sign Detection

**Figure 8:**
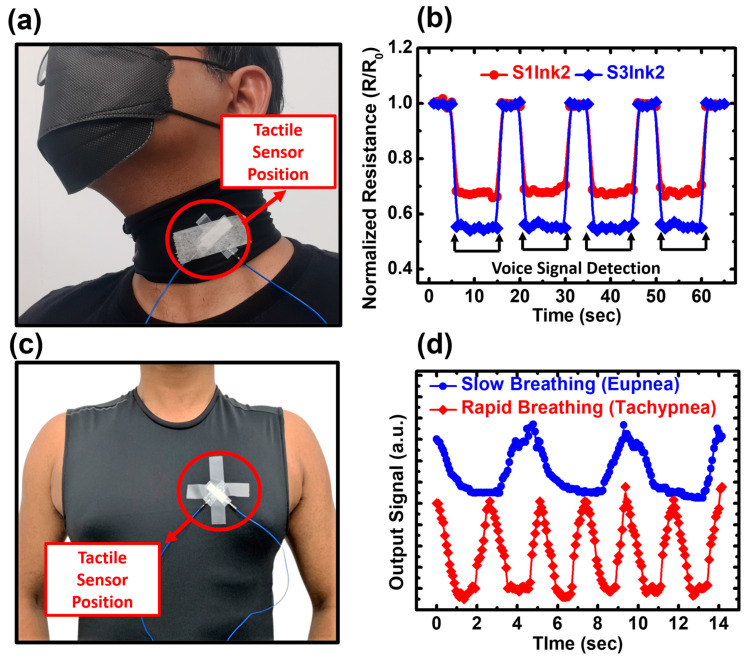
Demonstration of wearable applications to detect human vital signs (**a**) measurement setup of voice activity detection test in presence of background noise and (**b**) recorded speech pattern for 4 cycles. (**c**) Measurement setup of breathing pattern detection test and (**d**) recorded breathing pattern with two distinct natures i.e., eupnea and tachypnea.

The real-time applications of fabricated tactile sensors are then presented to explain the functionality and usability of our tactile sensor as a wearable device to detect human vital signs, which is suitable for both self-health monitoring and musical education. For the detection of human vital signs, two different applications, i.e., voice activity detection and breathing pattern detection, were executed with two Ink2-based tactile sensors with substrates S1 and S3. At first, the fabricated tactile sensor was used for voice activity detection (VAD) in the presence of background noise. Figure 8a shows the voice detection test setup. During speaking, a certain amount of vibration is generated, which is sensed by the highly sensitive sensor presented in this work. The tactile sensor was attached to a piece of fabric and it was wrapped around the throat. The sensor was placed in the particular position where the vibration is maximum. After firmly attaching the sensor, four verses from the famous poem “Stopping by Woods on a Snowy Evening” by American poet Robert Frost were recited. The average time required for each verse was 13 s. Figure 8b shows the obtained data for voice activity testing. It can be seen that both tactile sensors with S1 and S3 substrates successfully detected the vibrations generated during recitation and the measured data lies in the range of 0.1 and 0.2 kPa, which belongs to the subtle pressure range (approx. 0–0.5 kPa).

The sensors were further investigated by performing the detection of breathing patterns. The tactile sensor with an S3 substrate coated with Ink2 was used to distinguish two respiratory patterns, i.e., eupnea (slow/normal breathing) and tachypnea (rapid breathing). The tactile sensor was attached to the chest of a volunteer, as shown in Figure 8c; the variations of the electrical signals were monitored according to the inhalation and exhalation of the volunteer.

The result is graphically presented in Figure 8d. During inhalation, when the chest is expanded, the sensor can sense the pressure resulting from that. Hence, during inhalation, the output signal becomes low. Meanwhile, during exhalation when the chest returns to its original state, the pressure is released from the tactile sensor and the signal goes back to the initial high state. Two different breathing patterns were observed within a 14-s timeframe. When the volunteer was at resting position, the total number of breathing cycles was three within 14 s, which is within the normal range for eupnea (approx. 12–20 respirations per minute). The rapid breathing pattern detection was carried out immediately after 10 min of cardiovascular exercise (jogging and climbing stairs) done by the volunteer. The number of respiratory cycles within a 14-s timeframe was six, which matches the breathing rate of tachypnea (more than 20 respirations per minute). These real-time data prove that this low-cost tactile sensor has the potential to develop low-cost future wearable sensing devices to detect vital signs for health monitoring. By analyzing the voice activity in the presence of outside noise, this device can be used for the future diagnosis of voice disorders such as laryngitis, muscle tension dysphonia, vocal cord weakness, etc., whereas the breathing pattern detection data shows the potential of this device for the diagnosis of breathing disorders such as asthma or bronchitis.

#### 3.5.2. Posture Feedback during Guitar Playing Application

The tactile sensors were further used in the detection of strains from the tension of muscles while playing musical instruments. For this segment, a guitar was used as the musical instrument because of its worldwide popularity and acceptability. To play guitar, asymmetrical body postures and repetitive motions of the wrist and fingers are required and this often leads to musculoskeletal disorders such as over-use syndrome [58]. In one previous work, it was reported that 45% of injuries among guitarists were strongly tied to the hand and wrist [59]. Moreover, the possibility of injuries can also be caused by bad posture, too much pressure on the fretboard and bad finger technique. For example, putting too much pressure on the fretboard or holding the guitar pick with too much tension can develop into tendonitis, which can put a guitar player’s career in jeopardy [60].

**Figure 9:**
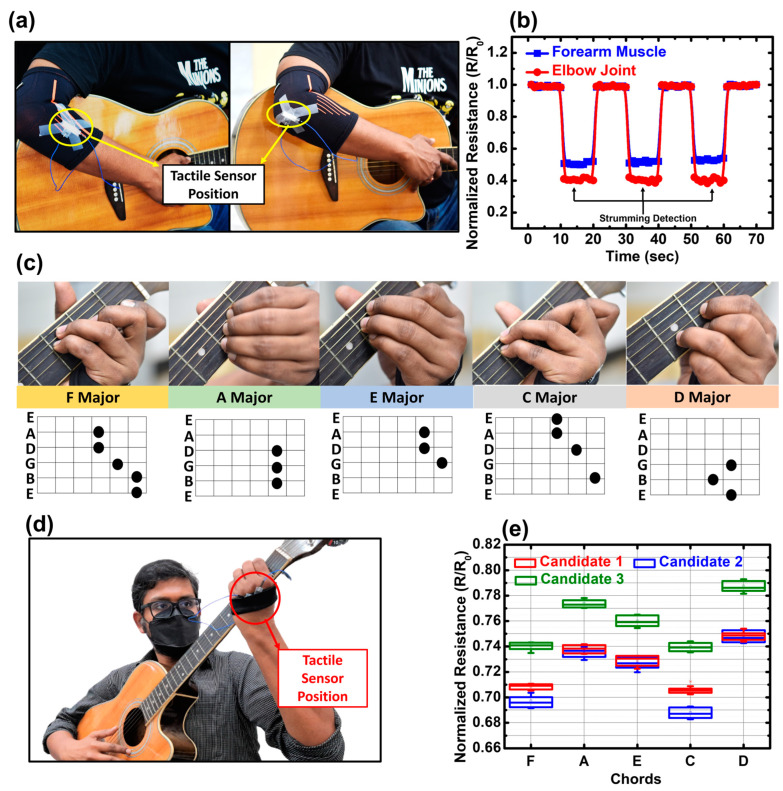
(**a**) Placement of tactile sensor on forearm and elbow joint with an arm wrap (**b**) obtained data for guitar strumming test (**c**) chord structure and holding pattern (**d**) measurement setup for chord progression detection test. The tactile sensor was attached with an arm wrap and worn around the left palm. The sensor was placed on the back side of the palm to detect the maximum strain while holding the chords (**e**) obtained data for three different candidates with different skill levels (beginner to professional).

Hence, the tactile sensor presented in this work can be a good candidate to monitor posture and muscle movement during playing. The tactile sensor with an S3 substrate coated with Ink2 was selected for this experiment. Two different experiments (detection of guitar strumming and chord progression) were carried out in this work while guitar playing. Figure 9a shows the experimental setup for guitar strumming detection. The tactile sensor was carefully attached to an arm wrap and worn to fix the sensor at two different positions on the right hand, i.e., forearm and elbow. The strain sensed by the tactile sensors during the back-and-forth movement of the right hand during strumming is graphically presented in Figure 9b. During movement of the right hand, an amount of strain was exerted on the forehand muscle that was relatively lower than the bending motion at the elbow joint. Hence, the applied strain at the tactile sensor was more at the elbow joint compared to the forearm muscle. However, the tactile sensors presented in this work were able to detect the pressure exerted by the forearm muscle successfully, which was equivalent to 0.2 kPa applied pressure.

For detecting the guitar chord progression, the sensor was attached in a smaller arm wrap and it was worn on the left palm. The chord structures and holding patterns are presented in Figure 9c. The tactile sensor was placed on the opposite of the palm, as shown in Figure 9d, since while holding a chord, the maximum amount of strain is exerted by the movement of fingers and muscles on that particular location of the left hand. This process is common for all guitar players with different palm shapes and sizes. Three volunteers with different skills were selected for this demonstration and they were asked to hold five different chords, i.e., F, A, E, C and D. They wore the arm wrap on their palm and it was confirmed that the tactile sensor attachment did not hamper their hand movement along the fretboard. When the guitar strings over the fretboard are held with the fingers in a particular manner, they form the chord, and based on the holding pattern the chords and the related sounds are different. Hence, it can be understood that with different holding patterns, the exerted strain on the back of the palm will be different, too. The difference in chords can be observed in Figure 9e. It can be understood that the D chord has a higher value of normalized ECR since it exerts the least amount of strain since it is one of the easiest chords to hold, whereas those chords that require stretching the fingers over a wide area of the fretboard exert high strain over the back of the palm, such as F and C, since both chords display a lower value of normalized ECR compared to others.

The data obtained from three volunteers with different playing skills are also presented. Candidate1 can be counted as an expert owing to her experience in guitar playing for more than 10 years; hence the data acquired from Candidate1 is considered as control data. Candidate2 has intermediary skills based on his experience of almost 2 years, whereas Candidate3 has a beginner level of skill since he has experience of 3 months. The data obtained for Candidate3 shows the highest value of normalized ECR value. In most cases, beginners struggle to apply the right amount of pressure over the strings. Hence, the exerted strain was low at the opposite of the palm, resulting in a higher normalized ECR for Candidate3, which proves the aforementioned statement. However, Candidate2 can hold the easier chords (D, A, and E) in the right manner since the data matches with the control data of Candidate1. However, the data for the F and C chords were lower than the data of Candidate1, which means that Candidate2 applied extra pressure over the strings to produce the right sound. This is a common problem for learners with intermediate skills since they intend to hold tougher chords perfectly and often apply extra pressure over the fretboard, which could cause fatigue over the left arm or even tendonitis. So, it is also necessary to learn how to relax the left hand while playing, which can be assisted by the tactile sensor presented in this work.

This sensor can also be used for other musical instrument education, such as violin or drums. With more work, this device can be developed further and it can be a game-changer in this present-day of online learning. With this device, learners will not be on their own because they will be able to track their progress successfully. Moreover, it could be possible to prevent the over-use syndromes, such as carpal tunnel syndrome and tendonitis, suffered by guitarists and other musicians by using the tactile sensor presented in this work to monitor the muscle strain during playing.

## 4. Discussion

In recent years, several works have reported the improvement of tactile sensing characteristics using skin-inspired resistive tactile–pressure sensors [9,19,21,23]. They all reported impressive sensitivity value, but some of their fabrication techniques to form these microstructures are complicated, and not quite feasible for mass production. The sensitivity value of the tactile sensor presented in this work belongs to the subtle pressure range, which is much lower than the reported pressure range of previous works. Most importantly, the sensor presented in this work showed a much quicker recovery time compared to others. The reported sensitivity values and recovery time of this work were further compared in Supplementary Table S1.

Additionally, this work demonstrates a huge improvement from our previous works, in which the tactile sensor was made on the PET-paper substrates with a sensitivity at 1%/kPa for approx. 0–0.05 MPa [35]. The sensitivity value was later improved to 1.04 kPa^−1^ with micropillar-structured graphene ink film as a sensing material and glass fiber as the substrate material [36]. A biodegradable PVA-based tactile sensor was reported with a sensitivity of 1.99 kPa^−1^ for 0.5 kPa [37]. With the porous cellulose fiber substrates, our tactile sensor was able to detect a very slight amount of pressure, i.e., 0.005 kPa, which is a significant development in this work. The sensitivity at 0.05 kPa pressure was greatly improved from 1%/kPa to 14.4 kPa^−1^ in this work. Moreover, the pressure range was improved to 5 kPa by incorporating porous cellulose fiber substrate in this work compared to our previously reported work. The details of these parameters are given in Supplementary Table S2.

Our proposed approach of using cellulose fiber substrates not only fully exploits the ECR sensing mechanism, but it shows high sensitivity by mimicking the microstructures of the spinosum layer between the dermis and epidermis of human skin. This skin-inspired structure was implemented over commercially available cellulose fiber substrates that possess similar elastic modulus values as human skin. The screen printing method has dominance in the printing sector owing to its simplicity, low cost, versatility and maturity. Though the goal of this skin-inspired tactile sensor was to detect the subtle pressure range, by doing careful optimization of the porous substrate, the tactile sensor can further be implemented for high-pressure sensing applications, too. Therefore, with this approach, the development of low-cost, flexible skin-inspired tactile sensors can give a cheaper and more mass-producible alternative for future applications, such as the detection of human vital signs and muscle movements.

The sensors successfully detected the voice activity in the presence of background noise, and even the vibration generated by voices with various pitches with further development. For example, if a person has a cough-related problem, then the voice-generated vibration will be different from the regular one. By analyzing the voice pattern, this device can be used for the early diagnosis of voice disorders such as laryngitis, muscle tension dysphonia, polyps or a cist on the vocal cord, etc. Also, both slow and rapid breathing patterns were identified by the paper-based tactile sensors. Within 14 s, the total number of respiratory cycles was three for slow breathing, which falls in the normal range for Eupnea (approx. 12–20 respirations per minute). For rapid breathing, the total number of respiratory cycles was 14 in a 14-s timeframe, which lies in the range of tachypnea (more than 20 respirations per minute). Hence, it can be understood that this device has the potential for the diagnosis of respiratory disorders such as asthma or bronchitis. With further development, this device can be a cheap alternative to existing vital-sign-detecting wearable systems that are currently available on the market.

This tactile sensor is also suitable for musical education. Very few works reported the usage of the tactile sensor assembly for musical education because, to analyze the progress of a learner, the sensor assembly needs to be sensitive enough to detect very small movements of the muscle, and the majority of the tactile sensors are designed to obtain sensitivity at a relatively higher pressure than the subtle pressure region. One of the common approaches to monitoring the learning process for online music education is the use of electromyography (EMG) to detect the signal generated by the forearm muscle as a gestural input interface to capture biofeedback signals found in the muscles during guitar playing [61,62]. This, however, is an indirect way of obtaining the signals. A similar result can be achieved with the very cheap alternative of the EMG armband, i.e., the tactile sensor presented in this work with high sensitivity. Moreover, it can be attached to different locations on the hand and the signals can be collected very easily. With more research, this sensor can be extended for other musical instruments (e.g., the violin) by attaching the sensors to different muscle locations to analyze the posture as a feedback module.

## 5. Conclusions

A skin-inspired flexible tactile sensor with interfacial microstructure has been developed over the cellulose fiber substrates to detect subtle pressure (approx. 0–0.5 kPa). The microstructures, which mimic the spinosum layer between the dermis and epidermis, were successfully made by using screen-printing methods for tactile perception by employing the electrical contact resistance change mechanism. The cellulose fiber substrates were further tested with different textures, compressibility (e.g., approx. 10^−4^–10^−1^ MPa), and porosity (e.g., 66%, 70%, 84% and 85%) for optimal tactile sensing performance. ECR variation at the interface after pressure application was the key mechanism for tactile sensing. The correlation between device performance and the substrate’s physical properties (compressibility, porosity) was also analyzed, and it was understood that high sensitivity can be obtained for the compressible and porous substrates. Multiple combinations of the inks and substrates were investigated and optimized to obtain a high sensitivity of 14.4 kPa^−1^ for low pressure of 0.05 kPa and a faster recovery time (approx. 2.5 ms). The larger deformation of compressible substrates was confirmed with the nanoindentation process, and it was explained in a theoretical model how it will impact the generation of electrical contact point generation at the interface. Our tactile sensors exhibited high sensitivity at lower pressure to detect human vital signs such as voice activity and breathing patterns. Moreover, the sensors with high sensitivity could distinguish the guitar strumming patterns and chord progression, showing the tremendous potential in the prevention of arm and wrist injuries such as tendonitis or over-use syndrome for guitar players.

## Figures and Tables

**Table 1:** Characteristics of Ink1 and Ink2.

Parameters	Ink1	Ink2
Thickness	12 µm	33.2 µm
Average RMS roughness	0.634 µm	0.999 µm
Cluster size	50 nm	96 nm
Viscosity at 1 S-1 shear rate	682.89 Pa.S	2549.14 Pa.S
Young’s Modulus	4.417 GPa	5.324 GPa
Hardness	0.094 GPa	0.151 GPa

## Data Availability

Data sharing is not applicable to this article.

## Supplementary Materials

The following supporting information can be downloaded at: https://www.mdpi.com/article/10.3390/bios13020174/s1, Figure S1: Resistive characteristics at lower pressure region of sensors with (a) Ink1 and (b) Ink2. The change of normalized resistance with applied pressure has been presented for the pressure range of 1 kPa.; Figure S2: (a) The normalized resistance deviation and (b) recovery time for fabricated tactile sensors.; Table S1: Device performance comparison between the reported work with the previous works.; Table S2: Device performance comparison between the reported work with the previous works done by our group.
