# Supplementary material for: Skin-Inspired Tactile Sensor on Cellulose Fiber Substrates with Interfacial Microstructure for Health Monitoring and Guitar Posture Feedback
Source: Biosensors (Basel). 2023 Jan 22;13(2):174. doi: 10.3390/bios13020174 (PMC9953271; doi:10.3390/bios13020174)
Supplement: Supplementary file 1 [file biosensors-13-00174-s001.zip › biosensors-2164270-supplementary.pdf]

biosensors MDPI

Supporting Information

Skin-Inspired Tactile Sensor on Cellulose Fiber Substrates with Interfacial Microstructure for Health Monitoring and Musical Education Applications

Rajat Subhra Karmakar [1], Chia-Pei Chu [2], Chia-Lin Li [3], Chun-Hway Hsueh [3], Ying-Chih Liao [2], and Yen-Wen Lu [1,*]

[1] Department of Biomechatronics Engineering, National Taiwan University, Taipei, Taiwan
[2] Department of Chemical Engineering, National Taiwan University, Taipei, Taiwan
[3] Department of Materials Science and Engineering, National Taiwan University, Taipei, Taiwan
* Correspondence: *Y.-W. Lu.: e-mail, yenwenlu@ntu.edu.tw; tel., +886-2-3366-5346

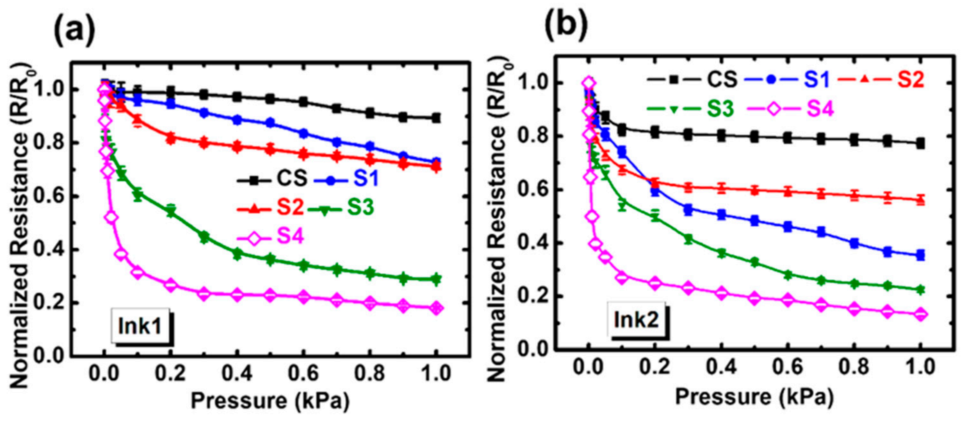

Figure S1: Resistive characteristics at lower pressure region of sensors with (a) Ink1 and (b) Ink2. The change of normalized resistance with applied pressure has been presented for the pressure range of 1 kPa.

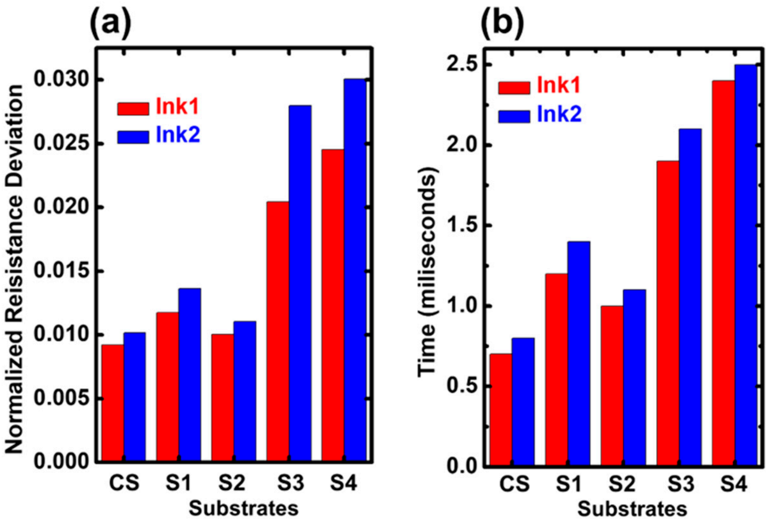

Figure S2: (a) The normalized resistance deviation and (b) recovery time for fabricated tactile sensors.

Supplementary Table S1. Device performance comparison between the reported work with the previous works.

Copyright: © 2023 by the authors. Licensee MDPI, Basel, Switzerland. This article is an open access article distributed under the terms and conditions of the Creative Commons Attribution (CC BY) license (https://creativecommons.org/licenses/by/4.0/).

**Device Details**	**Sensitivity**	**Sensing range**	**Recovery time**
Interlocked TPU@IL ionogel sensor.[1]	3.07 kPa^{-1}	0.01-1 kPa	62 ms
Flexible electronic skins based on interlocked microdome arrays.[2]	-15.1 kPa^{-1}	<0.5 kPa	~0.04 s
Pressure sensors with bionic spinosum microstructure with abrasive paper template and reduced graphene oxide.[3]	25.1 kPa^{-1}	0-2.5 kPa	80 ms
Flexible tactile sensor with skinlike dual-interlocked structure.[4]	0.0385 kPa^{-1}	0 - 497.6 kPa	400 ms
Screen-printed cellulose fiber tactile sensor **(Present Work)**.	14.4 kPa^{-1}	0-0.05 kPa	2.5 ms

In this table the sensitivity and recovery time of the cellulose fiber based tactile sensors were compared. It can be observed that all the aforementioned works have implemented an interlocked structure which were developed with very carefully. However, the complicated structure of these sensors are not practical enough for the mass production. Whereas, this present work has used an industry oriented fabrication method (screen printing) to develop a randomly distributed spinosum shaped microstructure based on the substrate roughness. The sensitivity at subtle pressure range presented in this work can not only be compared to the previous works but also it shows the clear detection ability of low pressure i.e., 0.005 kPa as shown in Figure S1. Most importantly the sensor presented in this work showed a rapid recovery time compared to the previous works.

Supplementary Table S2. Device performance comparison between the reported work with the previous works done by our group.

Device Details	Substrate	Sensing material	Pressure range	Sensitivity
PEDOT:PSS coated Tactile sensor (2013)[5]	Glass, PET an paper.	PEDOT:PSS	0-0.05 kPa	1%/kPa
Screen Printed Fabric Based Tactile sensor (2017)[6]	Fabric (glass fiber)	Silver Ink, Modified Graphene Ink	0-1 kPa	1.04 kPa^{-1}
PVA composite tactile sensor (2022)[7]	PVA Sheet	PVA-Fe PVA-MWCNT	0-0.5 kPa	1.99 kPa^{-1}
Screen-printed cellulose fiber tactile sensor **(Present work)**	Cellulose Fiber Substrate	Graphene Ink Carbon Black Ink	0-0.05 kPa	14.4 kPa^{-1}

In this table the improvement of our tactile sensors throughout the years has been summarized. In early stage the tactile sensor only showed 1%/kPa sensitivity for 0-0.05 kPa applied pressure. In this present work our fabricated tactile sensor has demonstrated a very high sensitivity of 14.4 kPa^{-1} at same pressure range (0.05 kPa) which is a significant improvement from our earlier works. Meanwhile the highest reported sensitivity at 1 kPa and 0.5 kPa are 1 and 1.7 kPa^{-1} respectively, which is close to the previously reported sensitivity values by us. However, the tactile sensor presented in this work with porous substrate has shown extended pressure range of 5 kPa which is higher than our previously reported work with PVA composite tactile sensor. Moreover, the presented tactile sensor in this work is able to detect a very low pressure of 0.005 kPa which was not been detected before by our previous tactile sensors. Hence it can be understood that the tactile sensor presented in this work has a tunable pressure range which suitable for both future low and high pressure sensing application.

References

1. Xu, Y.; Chen, L.; Chen, J.; Chang, X.; Zhu, Y. Flexible and Transparent Pressure/Temperature Sensors Based on Ionogels with Bioinspired Interlocked Microstructures. *ACS Appl. Mater. Interfaces* **2022**, *14*, 2122–2131, doi:10.1021/acsami.1c22428.
2. Park, J.; Lee, Y.; Hong, J.; Ha, M.; Jung, Y.-D.; Lim, H.; Kim, S.Y.; Ko, H. Giant Tunneling Piezoresistance of Composite Elastomers with Interlocked Microdome Arrays for Ultrasensitive and Multimodal Electronic Skins. *ACS Nano* **2014**, *8*, 4689–4697, doi:10.1021/nn500441k.
3. Pang, Y.; Zhang, K.; Yang, Z.; Jiang, S.; Ju, Z.; Li, Y.; Wang, X.; Wang, D.; Jian, M.; Zhang, Y.; et al. Epidermis Microstructure Inspired Graphene Pressure Sensor with Random Distributed Spinosum for High Sensitivity and Large Linearity. *ACS Nano* **2018**, *12*, 2346–2354, doi:10.1021/acsnano.7b07613.
4. Wang, Y.; Dai, S.; Mei, D.; Jin, J. A Flexible Tactile Sensor With Dual-Interlocked Structure for Broad Range Force Sensing and Gaming Applications. *IEEE Trans. Instrum. Meas.* **2022**, *71*, 1–10, doi:10.1109/TIM.2022.3147329.
5. Shiau, C.-C.; Liao, Y.-C.; Kao, Z.-K.; Yeh, Y.-C.; Lu, Y.-W. Paper-Based Flexible Taxel Device Using Electrical Contact Resistance Variation for Elasticity Measurement on Biological Objects. *IEEE Sens. J.* **2013**, *13*, 4038–4044, doi:10.1109/JSEN.2013.2271422.
6. Chen, W.-L.; Liao, Y.-C.; Lu, Y.-W. A Wearable Tactile Sensor Based on Electrical-Contact-Resistance (ECR) Variation with High Sensitivity for Health Monitoring. In Proceedings of the 2017 19th International Conference on Solid-State Sensors, Actuators and Microsystems (TRANSDUCERS); IEEE: Kaohsiung, June 2017; pp. 1116–1119.

7. Karmakar, R.S.; Chu, C.-P.; Liao, Y.-C.; Lu, Y.-W. PVA Tactile Sensors Based on Electrical Contact Resistance (ECR) Change Mechanism for Subtle Pressure Detection. *Sens. Actuator A Phys.* **2022**, *342*, 113613, doi:10.1016/j.sna.2022.113613.

Disclaimer/Publisher's Note: The statements, opinions and data contained in all publications are solely those of the individual author(s) and contributor(s) and not of MDPI and/or the editor(s). MDPI and/or the editor(s) disclaim responsibility for any injury to people or property resulting from any ideas, methods, instructions or products referred to in the content.
